# Serum Bilirubin Level Is Increased in Metabolically Healthy Obesity

**DOI:** 10.3389/fendo.2021.792795

**Published:** 2022-03-30

**Authors:** Jing Fu, Qiu Wang, Lin Zhang, Jia Liu, Guang Wang

**Affiliations:** Department of Endocrinology, Beijing Chao-Yang Hospital, Capital Medical University, Beijing, China

**Keywords:** bilirubin, obesity, metabolically benign, morbid, MHO

## Abstract

**Objectives:**

Bilirubin is a biochemical substance with metabolic benefits. The objective of this research was to elucidate the association between serum bilirubin levels and metabolic alterations in different obesity phenotypes.

**Methods:**

In total, 1,042 drug-naive participants were included in the study. Of them, 541 were obese patients and 501 were age-matched and sex-matched healthy control subjects. The obese patients were divided into metabolically healthy obesity (MHO) group and metabolically unhealthy obesity (MUHO) group according to the levels of fasting plasma glucose (FBG), triglyceride (TG), high-density lipoprotein cholesterol (HDL-C) and blood pressure (BP). Clinical and biochemical parameters including total bilirubin (TBil), indirect bilirubin (IBil) and direct bilirubin (DBil) were measured. ANOVA or Kruskal-Wallis H test was used to test differences among the three groups. Pearson and Spearman correlations were used to analyze the relationships between two parameters. The relationships between bilirubin and other variables were analyzed using Multivariate regression analysis.

**Results:**

MHO group had favorable blood pressure, glucose and lipids profiles, along with increased TBil and DBil, and decreased high-sensitivity C-reactive protein (hsCRP) and homeostasis model assessment of insulin resistance (HOMA-IR) levels when compared to MUHO group (*P* < 0.05 for all). TBil and DBil were negatively correlated with total cholesterol (TC), low-density lipoprotein cholesterol (LDL-C), fasting insulin (FINS), hsCRP and HOMA-IR, even after adjusted for age, gender and BMI (all *P* <0.01). Multivariate regression analysis demonstrated that HOMA-IR was independently correlated with TBil and DBIi levels (β = -0.400, *P* < 0.01).

**Conclusion:**

MHO group harbors increased bilirubin level compared with MUHO group. HOMA-IR was independently correlated with TBil and DBIi levels.

## Introduction

Obesity has become a global health problem due to its epidemic proportions and health hazard (1–3). Obesity is confirmed as one of the most important risk factors for dyslipidemia, type 2 diabetes and metabolic syndrome (1–3). Interestingly, a part of obese subjects show normal metabolism, which is defined as “metabolically healthy obesity (MHO)” (4, 5). However, exact mechanisms of MHO remain unclear.

Bilirubin, a product of heme metabolism, exerts anti-inflammatory and antioxidative effects (6, 7). Circulating bilirubin level is reported to be negatively correlated with the risk of cardio-metabolic diseases, including type 2 diabetes, NAFLD etc (7–11). As an intermediate state between health and metabolic disorders, MHO would manifest different bilirubin levels (12). However, the association between bilirubin and MHO is rarely reported.

The research aimed to elucidate the associations between serum bilirubin levels and metabolic parameters in different obesity phenotypes.

## Material and Methods

### Study Subjects

A total of 541 obese patients were consecutively enrolled in our study. The included patients were no less than 18 years, with BMI (body mass index) ≥ 28.0 kg/m^2^. All of the subjects received physical examinations in Beijing Chao-Yang Hospital, Capital Medical University between January 2018 and January 2019 (2). Meanwhile, 501 healthy individuals with normal weight (18.5 kg/m^2^ ≤ BMI < 24.0 kg/m^2^) were recruited as controls, and they were matched with obese cases in age and gender (2). The subjects had no evidences for alcohol abuse, cardiovascular disease, thyroid dysfunction, anemia, hematological disease, chronic hepatitis/cirrhosis, biliary obstruction disease, acute or chronic infections, renal insufficiency, systemic inflammation, or cancer. Individuals in pregnancy or taking medications known to influence bilirubin, liver function, insulin, glucose, lipid or blood pressure were excluded, such as glycyrrhizic acid, ursodeoxycholic acid, potassium magnesium aspartate, “Yinzhihuang” granules, Metformin, Reserpine, Guanethidine, etc. In addition, persons meeting any one of the following conditions were also removed: serum bilirubin levels ≥ 2 times of upper limit of normal (ULN) (ULNs: TBIL: 21.0 *μ*mol/L and DBIL : 6.8 *μ*mol/L) or serum ALT and/or AST and/or GGT levels ≥ 3ULN (ULNs: alanine aminotransferase (ALT) : 50 U/L, aspartate aminotransferase (AST) : 40 U/L, and gamma-glutamyl transferase (GGT) : 60 U/L).

When obese individuals (BMI ≥28 kg/m^2^) did not conform to the following criteria, they were categorized as MHO (13): (1) elevated FBG (≥5.6 mmol/L), (2) elevated TG (≥1.7 mmol/L), (3) reduced HDL-C (<1.0 mmol/L for men and <1.3 mmol/L for women), and (4) elevated SBP (≥130 mmHg) or/and DBP (≥85 mmHg). Obese patients who had one or more of these four metabolic risk components were categorized as metabolically unhealthy obesity (MUHO) (13).

This research was performed according to the Declaration of Helsinki ethical principles. Ethics approval was given by the Institutional Review Board (IRB) of Beijing Chao-Yang Hospital, Capital Medical University. Written informed consents were obtained from all subjects.

### Clinical and Biochemical Indicators’ Measurement

Both health status and medical history were collected through a standard questionnaire during a face-to-face interview, including alcohol consumption, medication status, physical activity, and history of diseases. Height was measured, with an accuracy of 0.1 cm, and weight was accurate to 0.1 kg, by professional medical staff, while participants were wearing light clothing without shoes. Body mass index (BMI) was calculated as weight/body height (kg/m^2^). We employed a sphygmomanometer to measure sitting blood pressure for non-dominant arm after at least ten-minute rest.

After overnight fasting, blood samples were collected from median cubital vein. All biochemical indicators were measured using an automatic biochemical analyzer (Hitachi 747, Roche Diagnostics, Germany), except for fasting plasma insulin (FINS), glycated hemoglobin A1c (HbA1c) and high-sensitivity C-reactive protein (hsCRP). Alanine aminotransferase (ALT), aspartate aminotransferase (AST) and gamma-glutamyl transpeptidase (GGT) were measured *via* velocity method. Total bilirubin (TBil) and direct bilirubin (DBil) were measured through vanadate oxidation method. Indirect bilirubin (IBil) was calculated based on TBil minus DBil. Total cholesterol (TC), triglyceride (TG), high-density lipoprotein cholesterol (HDL-C) and low-density lipoprotein cholesterol (LDL-C) were assessed adopting colorimetric enzymatic methods. Plasma TC and TG were tested employing enzymatic cholesterol oxidase reaction and glycerol lipase oxidase reaction, respectively. HDL-C and LDL-C were assessed *via* direct measurement. Fasting plasma glucose (FBG) were estimated utilizing glucose oxidase assay. FINS concentrations were tested using chemiluminescence assay (Dimension Vista, Siemens Healthcare Diagnostics, Germany). HbA1c was measured applying high-performance liquid chromatography (HPLC) on an automatic biochemical analyzer (HLC-723G7, Tosoh Corporation, Tokyo, Japan). hsCRP levels were determined with immunonephelometric analysis. To estimate insulin resistance, homeostasis model assessment of insulin resistance (HOMA-IR) was calculated: HOMA-IR = [FINS (μIU/mL) * FBG (mmol/L)/22.5] (14).

### Statistical Analysis

All statistical analyses were performed using SPSS 21.0 (SPSS, Chicago, IL, USA). Continuous variables in normal distribution were recorded as mean values ± standard deviation (SD). Because values for ALT, GGT, TG, FINS, hsCRP and HOMA-IR did not conform to normal distribution, they were represented by the median, 25% quartile and 75% quartile. Chi-squared test was adopted to analyze categorical variables. ANOVA or Kruskal-Wallis H test was used to compare differences among the three groups. *Post hoc* analyses were performed. The relationships between two parameters were analyzed using Pearson and Spearman correlations. The relationship between bilirubin and other variables was analyzed using Multivariate regression analysis. *P* < 0.05 (two-tailed) revealed statistical significance of results.

## Results

### Baseline Characteristics of control, MHO and MUHO Groups

Baseline characteristics of the included subjects were summarized in **Table 1**. Analysis results showed that age and gender were similar among control, MHO and MUHO groups. Among these three groups, no significant differences were observed in serum IBil levels. Significant differences were identified in BMI, SBP, DBP, ALT, AST, GGT, TBil, DBil, TC, TG, HDL-C, LDL-C, FBG, FINS, HbA1c, hsCRP and HOMA-IR levels among control, MHO and MUHO groups (all *P* < 0.01).

**Table 1:** Baseline characteristics of control, MHO and MUHO groups.

Parameters	Control group (n = 501)	MHO group (n = 51)	MUHO group (n = 490)	*P*
Age,y	36.7 ± 9.0	36.7 ± 8.8	36.7 ± 7.5	.998
Gender, M/F, n	418/83	9/42	410/80	.965
BMI, kg/m^2^	22.06 ± 1.61	30.03 ± 1.66^**^	30.40 ± 2.20^**^	.000
SBP, mmHg	116.9 ± 8.2	121.3 ± 5.3	132.0 ± 12.89^**^^$$^	.000
DBP, mmHg	71.7 ± 7.6	73.7 ± 6.3	81.6 ± 10.1^**^^$$^	.000
ALT, U/L	20.0 (16.0 – 28.0)	24.0 (20.5 – 35.0)	37.5 (29.0 – 57.2)^**^	.000
AST, U/L	20.1 ± 6.4	21.5 ± 4.6	25.6 ± 9.23^**^	.000
GGT, U/L	19.0 (14.0 – 27.0)	24.0 (19.5 – 43.0)	40.0 (26.7 – 57.2)^*^	.000
TBil, μmol/L	16.78 ± 4.01	15.29 ± 3.92	14.70 ± 3.67**^$^	.002
DBil, μmol/L	5.90 ± 1.87	5.30 ± 1.89	4.72 ± 1.80^**^^$$^	.000
IBil, μmol/L	10.88 ± 3.17	9.98 ± 3.54	9.97 ± 3.07	.076
TC, mmol/L	4.70 ± 0.83	4.82 ± 0.91	5.21 ± 1.08^**^	.000
TG, mmol/L	0.96 (0.75 – 1.19)	1.03 (0.75 – 1.26)	2.04 (1.46 – 2.91)^**^^$$^	.000
HDL-C, mmol/L	1.33 ± 0.25	1.21 ± 0.16	1.03 ± 0.24^**^^$$^	.000
LDL-C, mmol/L	2.74 ± 0.68	2.98 ± 0.70	2.99 ± 0.73^**^	.002
FBG, mmol/L	5.19 ± 0.22	5.18 ± 0.30	5.74 ± 1.12^**^^$$^	.000
FINS, mIU/L	9.00 (6.41 – 13.01)	15.22 (12.20 – 16.90)^*^	19.95 (15.73 – 31.52)^**^	.000
HbA1c, %	5.27 ± 0.17	5.35 ± 0.49	6.08 ± 0.64^*^^$^	.000
hsCRP, mg/L	0.19 (0.08 – 0.81)	0.82 (0.10 – 2.57)^*^	3.94 (1.49 – 6.81)^**^^$$^	.000
HOMA-IR	2.01 (1.48 – 2.66)	3.50 (2.71 – 4.00)^*^	5.06 (3.71 – 7.85)^**^^$$^	.000
Metabolic components				
Elevated BP^a^	0	0	286 (58.4%)	
Hyperglycemia^b^	0	0	226 (46.1%)	
Dyslipidemia^c^	0	0	397 (81.0%)	

Data are means ± standard deviation unless indicated otherwise. ALT, GGT, TG, FINS, hsCRP, and HOMA-IR are shown as medians (upper and lower quartiles). MHO, metabolically healthy obesity; MUHO, metabolically unhealthy obesity; M, males; F, females; BMI, body mass index; SBP, systolic blood pressure; DBP, diastolic blood pressure; ALT, alanine aminotransferase; AST, aspartate aminotransferase; GGT, gamma-glutamyl transpeptidase; TBil, total bilirubin; DBil, direct bilirubin; IBil, indirect bilirubin; TC, total cholesterol; TG, triglyceride; HDL-C, high-density lipoprotein cholesterol; LDL-C, low-density lipoprotein cholesterol; FBG, fasting blood glucose; FINS, fasting insulin; HbA1c, hemoglobin A1c; hsCRP, high-sensitivity C-reactive protein; HOMA-IR, homeostasis model assessment of insulin resistance.

*comparison between MUHO group and control group, *P < 0.05, **P < 0.01.

^$^comparison between MUHO group and MHO group, ^$^P < 0.05, ^$$^P < 0.01.

a Elevated BP were defined as, elevated SBP (≥130 mmHg) or/and DBP (≥85 mmHg);

b Hyperglycemia, elevated FBG (≥5.6 mmol/L);

c Dyslipidemia, elevated TG (≥1.7 mmol/L) or/and reduced HDL-C (<1.0 mmol/L for men and <1.3 mmol/L for women).

Further *post hoc* analysis showed that both of MHO and MUHO groups had significantly increased BMI, FINS, hsCRP and HOMA-IR compared with control group (**Table 1**). Moreover, patients in MUHO group had significantly increased SBP, DBP, ALT, AST, GGT, TC, TG, LDL-C, FBG and HbA1c levels, and decreased Tbil, DBil and HDL-C levels when compared to healthy controls (**Table 1**). MHO and MUHO groups showed similar tendencies in BMI, ALT, AST, GGT, IBil, TC, LDL-C and FINS levels (**Table 1**). Interestingly, MHO group had relatively lower SBP, DBP, TG, FBG, HbA1c, hsCRP and HOMA-IR levels, and higher TBil, DBil and HDL-C levels than MUHO group (**Table 1**).

### Comparison on Bilirubin Levels Among Control, MHO and MUHO Groups

We compared TBil, DBil and IBil levels among control, MHO and MUHO groups. As displayed in **Supplementary Figure 1**, MUHO group showed significantly lower level of TBil than control (*P*<0.01) and MHO (*P*<0.05) groups. MHO and control groups showed similar TBil values (*P*>0.05). As for IBil level, there were no significant differences among control, MHO and MUHO groups (*P*>0.05 for all) (**Supplementary Figure 2**). In addition, the level of DBil was decreased in MUHO group, compared with control and MHO groups (*P*<0.01 for both). Meanwhile, control and MHO groups showed insignificant difference in DBil level (*P*>0.05) (**Supplementary Figure 3**).

### The Associations Between Bilirubin and Clinical Parameters in All Subjects

TBil level showed negative association with BMI, GGT, TC, TG, LDL-C, FINS, hsCRP and HOMA-IR, and positive association with HDL-C level (**Table 2**). Moreover, these significant correlations did not show remarkable alteration after adjusted for age, gender and BMI (*P <*0.01 for all).

**Table 2:** Correlation between bilirubin and clinical parameters in all participants.

Parameters	TBil		DBil		IBil	
	r	P	*r*	*P*	*r*	*P*
Age	.016	.616	-.069	.030	.057	.075
BMI	-.082	.010	-.134	.000	-.052	.104
SBP	-.006	.842	-.034	.286	.008	.812
DBP	-.022	.482	-.029	.370	.046	.148
ALT	-.036	.261	-.098	.002	-.003	.920
AST	.019	.550	.015	.631	.020	.535
GGT	-.073	.022	-.087	.006	-.010	.751
TC	-.078	.014	-.352	.000	.060	.059
TG	-.089	.005	-.291	.000	-.017	.592
HDL-C	.138	.000	.117	.000	.140	.000
LDL-C	-.030	.347	-.229	.000	.069	.030
FBG	-.043	.174	-.089	.005	-.018	.566
FINS	-.206	.000	-.236	.000	-.176	.000
HbA1c	-.158	.122	-.194	.057	-.132	.196
hsCRP	-.364	.000	-.408	.000	-.313	.000
HOMA-IR	-.205	.000	-.244	.000	-.171	.000

BMI, body mass index; SBP, systolic blood pressure; DBP, diastolic blood pressure; ALT, alanine aminotransferase; AST, aspartate aminotransferase; GGT, gamma-glutamyl transpeptidase; TC, total cholesterol; TG, triglyceride; HDL-C, high-density lipoprotein cholesterol; LDL-C, low-density lipoprotein cholesterol; FBG, fasting blood glucose; FINS, fasting insulin; HbA1c, hemoglobin A1c; hsCRP, high-sensitivity C-reactive protein; HOMA-IR, homeostasis model assessment of insulin resistance; TBil, total bilirubin; DBil, direct bilirubin; IBil, indirect bilirubin.

Serum DBil level was negatively correlated with age, BMI, ALT, GGT, TC, TG, LDL-C, FBG, FINS, hsCRP and HOMA-IR, and positively associated with HDL-C levels (**Table 2**). After adjusted for age, gender and BMI, the associations of DBil level with TC, LDL-C, FINS, hsCRP and HOMA-IR were still significant (TC: *r* = - 0.493; LDL-C: *r* = - 0.530; FINS: *r* = - 0.441; hsCRP: *r* = - 0.335; HOMA-IR: *r* = - 0.380; all *P <*0.01).

Serum IBil level was negatively correlated with hsCRP and HOMA-IR, and positively correlated with HDL-C and LDL-C levels (**Table 2**). However, these associations were insignificant after adjusted for age, gender and BMI.

### Multivariate Stepwise Regression Analysis on Relationships Between Serum Bilirubin Level and Other Clinical Parameters

Multivariate regression analysis was used to evaluate the correlation between serum bilirubin level and clinical and biochemical parameters, including age, gender, BMI, TG, FBG, hsCRP and HOMA-IR. Results showed HOMA-IR was independently correlated with TBil and DBil levels (*β* = -0.400, *P* < 0.01).

## Discussion

Compared with MUHO group, MHO group had favorable blood pressure, glucose and lipid profiles, increased TBil and DBil levels, and decreased hsCRP and HOMA-IR levels, even in a comparable BMI level. Serum TBil and DBil levels were negatively correlated with TC, LDL-C, FINS, hsCRP and HOMA-IR. HOMA-IR was independently correlated with TBil and DBil levels.

Apart from favorable metabolic parameters, MHO group had decreased hsCRP and HOMA-IR levels when compared to MUHO group, even in a comparable BMI level. Insulin resistance is a major mechanism for metabolic syndrome (15, 16). Chronic overfeeding induces adipocyte hypertrophy, and then activates inflammatory pathways and accelerates inflammatory cells’ infiltration in adipose tissue, which further promote chronic low-grade inflammation and systemic insulin resistance in diet-induced obese mice (15–17). A recent human study showed that serum bilirubin level was negatively associated with inflammatory cytokines such as TNF-α, IL-6 and CRP, and positively associated with anti-inflammatory adiponectin (18). Consistently, our study showed that relatively reduced inflammatory state and heightened insulin sensitivity predicted favorable metabolic parameters in MHO patients.

Bilirubin has been recognized as a biochemical substance with metabolic benefits in recent years (19). Bilirubin includes two subtypes: DBil and IBil. DBil could be converted from IBil by UDP-glucuronyl transferase 1A1 (UGT1A1) in liver. The present research displayed that MHO patients had higher Tbil and DBil levels compared with MUHO group. Moreover, serum DBIL level was negatively correlated with HOMA-IR level. Multivariate regression analysis displayed that HOMA-IR was independently correlated with TBil and DBil levels. Consistently, previous studies showed that higher plasma TBil and DBil levels were associated with better metabolic parameters and lower risk of NAFLD (10, 20, 21). Morbid obesity decreases UGT1A1 activity, which may lead to low DBil level (22). In our study, both of MHO and MUHO groups showed decreased TBil and DBil levels. Moreover, IBil level in MHO group was similar to that in MUHO group. So significant down-regulation of TBil level in MUHO group could be attributed to decreased DBil level caused by UGT1A1 defect. Bilirubin administration for 14 days significantly reduced body weights, improved glucose tolerance and elevated insulin sensitivity in DIO mice (23). Bilirubin treatment reduced macrophage infiltration, and inhibited the expressions of TNF-α, IL-1β and MCP-1 in adipose tissue of diet-induced obese mice (24). Bilirubin also regulated T helper type 17 (Th17) immune responses and inhibited the generation of ROS induced by toll-like receptor 4 (25, 26). In the present study, correlation analysis found that serum DBil level was negatively associated with hsCRP level. These findings from our study and previous ones suggested that increased TBil and DBil levels predicted normal metabolism among obese subjects. In normal physiological pH condition, bilirubin is a fat soluble substance difficult to dissolve in water. In blood, bilirubin binds to albumin for transportation. It was reported that DBil was weakly bound to albumin, while IBil was strongly bound to albumin (27). So DBil might be easily separated from albumin, and played protective roles in metabolic processes.

In addition, serum levels of TBil and DBil were negatively correlated with atherogenic blood lipids (TC and LDL-C). In diet-induced obese mice, bilirubin treatment significantly reduced TC level, accompanied by reduced hepatic expression of SREBP-1, a factor required for *de novo* lipogenesis (23). Consistently, patients with Gilbert’s syndrome had reduced TC and LDL-C levels when compared to matched controls (19, 28, 29). Bilirubin could promote lipid catabolism and inhibit lipid accumulations (30, 31). Bilirubin might bind to PPARα and promote β-hydroxybutyrate, and then activate hepatic β-oxidation pathway, thus boosting lipid metabolism (32, 33).

Several limitations in our research should be acknowledged here. First, this cross-sectional study cannot render causal inferences. Besides, Gilbert’s syndrome, caused by reduced activity of UGT enzyme, is a common hereditary disease featured by hyperbilirubinemia. Although participants with serum bilirubin level ≥ 2ULN were excluded, Gilbert’s syndrome cases with serum bilirubin levels ≤ 2ULN might be recruited due to the absence of genetic examination. Finally, we only observed the association between bilirubin and metabolic parameters, and precise mechanism was not explored. In addition, only 51 patients were included in MHO group that might reduce statistical power of our analysis. Moreover, the prevalence of MHO was 9.43% in our study population, which was lower than previously reported occurrence rate (10%-30%) (5). Selection bias might contribute to this difference. More prospective and larger-scale studies are needed to determine the function and mechanisms of bilirubin in the progression of metabolic disorders in obese patients.

## Conclusions

Comparing with MUHO group, MHO group has favorable blood pressure, glucose and lipid profiles, apart from increased TBil and DBil levels and decreased hsCRP and HOMA-IR levels. Multivariate regression analysis shows that HOMA-IR is independently correlated with TBil and DBil levels.

## Data Availability Statement

The original contributions presented in the study are included in the article/**Supplementary Material**. Further inquiries can be directed to the corresponding author.

## Ethics Statement

The studies involving human participants were reviewed and approved by the Institutional Review Board (IRB) of Beijing Chao-Yang Hospital, Capital Medical University. The patients/participants provided their written informed consent to participate in this study.

## Author Contributions

JF, QW, and LZ conceived and designed the experiments, analyzed the data, and wrote the paper. JL and GW performed the experiments. All authors contributed to the article and approved the submitted version.

## Funding

This work was supported by grants from the Beijing Talents foundation [2018-12] to JL, and the Chinese National Natural Science Foundation [No. 81770792] and Key Projects of Science and Technology Planning of Beijing Municipal Education Commission [KZ201810025038] to GW. The funders had no role in study design, data collection and analysis, decision to publish, or preparation of the manuscript.

## Conflict of Interest

The authors declare that the research was conducted in the absence of any commercial or financial relationships that could be construed as a potential conflict of interest.

## Publisher’s Note

All claims expressed in this article are solely those of the authors and do not necessarily represent those of their affiliated organizations, or those of the publisher, the editors and the reviewers. Any product that may be evaluated in this article, or claim that may be made by its manufacturer, is not guaranteed or endorsed by the publisher.
