# Supplementary material for: Serum Bilirubin Level Is Increased in Metabolically Healthy Obesity
Source: Front Endocrinol (Lausanne). 2022 Mar 30;12:792795. doi: 10.3389/fendo.2021.792795 (PMC9005889; doi:10.3389/fendo.2021.792795)

Supplementary figure legends

Supplementary figure 1. Serum TBil levels of the control, MHO and MUHO groups. TBil: total bilirubin; MHO: metabolically healthy obesity; MUHO: metabolically unhealthy obesity; NS: not significant.

Supplementary figure 2. Serum IBil levels of the control, MHO and MUHO groups. IBil: indirect bilirubin; MHO: metabolically healthy obesity; MUHO: metabolically unhealthy obesity; NS: not significant.

Supplementary figure 3. Serum DBil levels of the control, MHO and MUHO groups. DBil: direct bilirubin; MHO: metabolically healthy obesity; MUHO: metabolically unhealthy obesity; NS: not significant.

Supplementary figure 1.

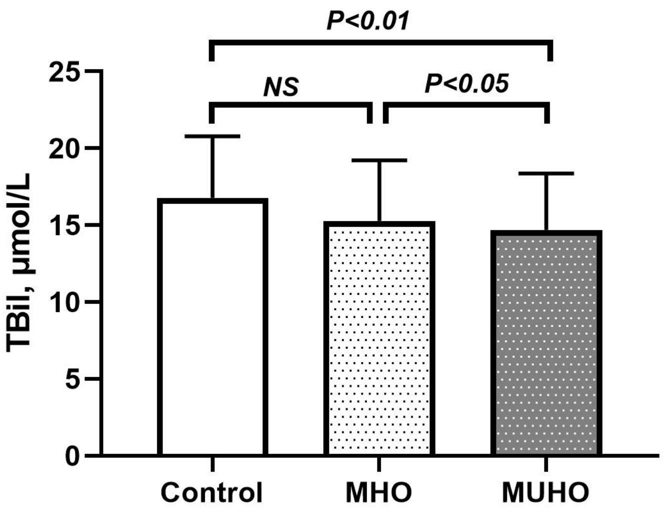

Supplementary figure 2.

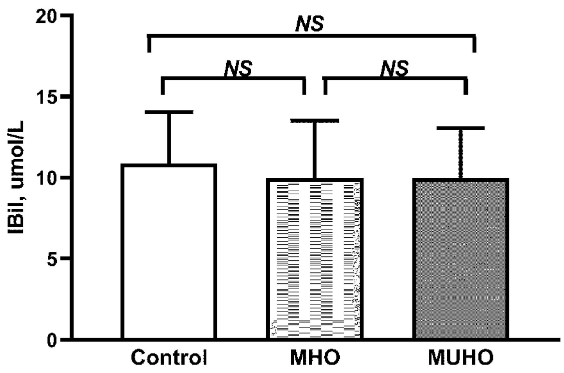

Supplementary figure 3.

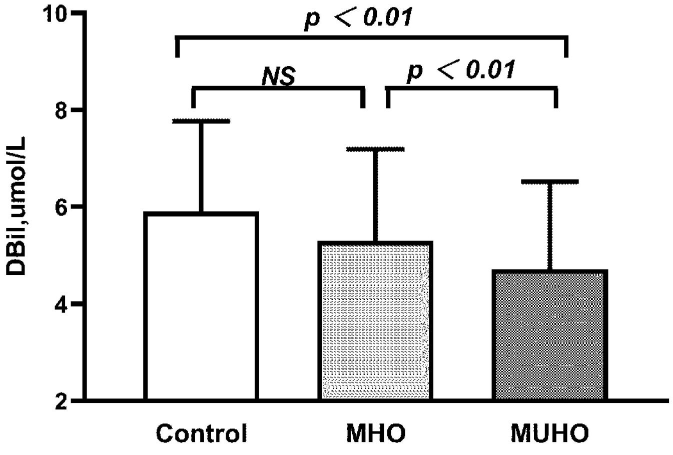

Supplement: Supplementary file 1 [file DataSheet_1.pdf]
